# Simultaneous Bilateral Total hip Arthroplasty in Patients With Juvenile Idiopathic Arthritis via Direct Anterior Approach: Long-Term Outcomes

**DOI:** 10.1016/j.artd.2024.101557

**Published:** 2024-10-29

**Authors:** Mohammadreza Razzaghof, Mohammad Vahedian Ardakani, Mohammad Poursalehian, Seyyed Hossein Shafiei, Mahlisha Kazemi, Seyed Mohammad Javad Mortazavi

**Affiliations:** a Joint Reconstruction Research Center, Tehran University of Medical Sciences, Tehran, Iran; b Department of Orthopedic Surgery, Imam Khomeini Hospital Complex, Tehran University ofMedical Sciences, Tehran, Iran; c Orthopedic Surgery Research Center, Sina University Hospital, Tehran University of MedicalSciences, Tehran, Iran; d Department of Orthopedics, Shariati Hospital, Tehran University of Medical Sciences, Tehran, Iran

**Keywords:** Juvenile idiopathic arthritis (JIA), Total hip arthroplasty (THA), Direct anterior approach (DAA), Bilateral hip involvement, Functional outcomes

## Abstract

**Background:**

Juvenile idiopathic arthritis (JIA) often results in significant bilateral hip damage, necessitating total hip arthroplasty (THA). Simultaneous bilateral THA offers potential advantages, particularly when executed via the Direct Anterior Approach (DAA). This study aims to assess the functional, radiological, and patient-reported outcomes, along with the complications of bilateral uncemented THA performed via DAA in patients with JIA.

**Methods:**

A retrospective review of 39 patients with JIA who underwent bilateral THA via DAA from January 2006-January 2015 was conducted. Inclusion and exclusion criteria were defined, focusing on a minimum of 7 years of post-THA follow-up. Functional outcomes were assessed using the Harris Hip Score.

**Results:**

Data were available for 33 patients (66 hips). The mean age at surgery was 21.3 years, and the average follow-up was 11.3 years. All patients reported severe bilateral hip pain presurgery, which was alleviated post-THA. The mean preoperative Harris Hip Score improved from 49.6-79.7 postoperatively. Complications included 3 calcar cracks, 2 greater trochanter fractures, and 1 superficial wound dehiscence. No instances of dislocation, postoperative periprosthetic fracture, or any revision surgery were recorded.

**Conclusions:**

Simultaneous bilateral THA using DAA is an effective and safe surgical approach for patients with JIA with bilateral end-stage hip involvement, providing notable improvements in functional and radiological outcomes while maintaining a favorable complication profile.

**Level of evidence:**

IV.

## Introduction

Juvenile idiopathic arthritis (JIA) is the most common form of arthritis in children, with an annual incidence rate of approximately 1 in 10,000 children [1]. Notably, JIA's manifestation tends to differ from adult rheumatoid arthritis, with severe JIA often causing significant impact on the hips [2]. For severe joint destruction resulting from JIA, total joint replacement is usually considered [3]. Total hip arthroplasty (THA) often faces challenges due to the higher activity levels, demanding physical requirements, and repetitive hip loading common in young patients, leading to an accelerated failure rate [4].

Despite these challenges, recent studies with mid- to long-term follow-up have indicated acceptable outcomes for THA in these young patients [5]. Of note, JIA typically affects both hips, necessitating bilateral THA [6]. In clinical practice, while staged bilateral THA is often employed, the use of simultaneous bilateral THA could present significant advantages [7]. These benefits include reduced anesthetic time, shorter hospital stays, and an expedited rehabilitation process, which contribute to overall cost-effectiveness. Among the various surgical approaches, the direct anterior approach (DAA) is often preferred for simultaneous procedures [8]. The DAA is associated with reduced intraoperative bleeding, eliminates the need for position change during the 2 stages, and tends to cause less muscle damage [9,10]. Consequently, these factors facilitate quicker recovery, shorten the rehabilitation period, and allow for more accurate measurements of limb length and offset during the surgery. One complication often encountered in these patients is the presence of flexion contracture, which can pose difficulties during the rehabilitation process [11,12]. However, this issue can be substantially mitigated intraoperatively through the anterior capsulectomy, that is, integral to the DAA surgical technique.

Through our practice we have been using simultaneous bilateral THA for patients with JIA. To date, no studies have reported on the long-term outcomes of bilateral THA using DAA in patients with JIA, who are typically younger than other patients requiring this procedure. Therefore, this study aims to evaluate the radiological and clinical outcomes of bilateral uncemented THA performed via DAA in patients with JIA with bilateral hip involvement.

## Methods

For this study, we accessed our institutional database to retrospectively identify 39 consecutive patients with JIA presenting with bilateral end-stage hip involvement. These patients had undergone bilateral THA through DAA between January 2006 and January 2015. Patient recruitment required a minimum of 7 years of follow-up.

Inclusion criteria for this study encompassed patients diagnosed with JIA presenting with bilateral end-stage hip involvement, who had undergone bilateral THA through DAA between January 2006 and January 2015, with a minimum of 5 years since the onset of JIA and at least 7 years post-THA. Additionally, eligible participants were required to have complete medical records and radiographs available for review and demonstrate a willingness to participate in physical examinations and phone interviews.

The exclusion criteria included:

a) Patients who underwent THA using surgical approaches other than DAA
b) patients with incomplete or unavailable medical records and radiographs,
c) patients unwilling to participate in physical examinations and phone interviews, and
d) patients noncompliant with the postoperative protocol,
e) hip involvement due to etiologies other than JIA,
f) less than 5 years elapsed since the onset of JIA, and
g) less than 7 years elapsed post-THA.

The data collection process involved reviewing medical records and radiographs, conducting physical examinations, and phone interviews carried out by 2 investigators not involved in the surgeries. Patients were questioned about their pain, daily movement limitations, activities, and quality of life. Furthermore, we evaluated operation times, intraoperative and postoperative complication rates, functional outcomes, and average hospital stay durations. The Harris Hip Score (HHS) was used to evaluate clinical outcomes for total hip arthroplasties. HHS data were collected both preoperatively and at the final follow-up.

All procedures were performed using spinal anesthesia, with patients in a supine position. Surgical intervention involved a modified Smith-Peterson DAA, complete anterior capsulectomy, double neck osteotomy, and soft tissue release performed for all hips. Details regarding the types of prosthesis used can be found in Table 1. The differences in the used stems were based on the availability of prosthesis at the time of surgery.

**Table 1:** Used prosthesis details.

Component	Type/model	Quantity	Size range	With screws
Cementless cup	Trilogy	42	42-56	39
	Continuum	10	44-54	4
	Pinnacle	12	44-52	9
	SecureFit PSL	2	52	2
Bearing surface	Metal on Highly Cross-Linked Polyethylene	66	-	-
Femoral stems	Fitmore	30	-	-
	Corail	24	-	-
	Wagner stem (small cone size)	12	-	-
Femoral heads	-	24	28 mm	-
	-	6	32 mm	-
	-	36	36 mm	-

Following surgery, no suction drains were used. Patients were administered a first-generation cephalosporin (Cefazolin) for 24 hours as infection prophylaxis and oral anticoagulation (aspirin) for 6 weeks. According to our standardized postoperative protocol, all patients except 9 were permitted weight-bearing as tolerated with crutches on the day following surgery. Five of them had fractures. Four of them had very poor bone quality, and to prevent fractures, they were advised partial weight-bearing at the surgeon's discretion.

### Statistical analyses

HHS change before surgery and at the latest follow-up were analyzed using a *t* test, with a *P* value of less than .05 deemed statistically significant. All statistical analyses were performed using SPSS v.22 (Chicago Inc., Chicago, IL).

## Results

Of the 39 patients diagnosed with JIA who underwent bilateral THA, data was available for 33 individuals (66 hips). The reasons for unavailability were as follows: 1 patient was unresponsive to phone contact, 2 declined radiographic evaluation, and 3 lacked preoperative data. Of the 33 individuals, 21 were female and 12 were male. The mean age at the time of surgery was 21.3 years, ranging from 14-31 years. On average, patients underwent hip replacement 13.2 years (range 5-17.5 years) after disease onset. The mean body mass index was 18.1 (range 17.1-23.4). The mean follow-up of patients was 11.3 years (range: 7-16) (Table 2).

**Table 2:** Demographic information and clinical characteristics of the study population.

Characteristic	Mean (range) or number
Total patients	33
Female	21
Male	12
Age at time of surgery (y)	21.3 (14-31)
Time from disease onset to surgery (y)	13.2 (5-17.5)
Body mass index (BMI)	18.1 (17.1-23.4)
Follow-up period (y)	11.3 (7-16)
Patients with knee involvement	24 (18 bilateral)
Patients with ankle involvement	15
Patients with small joint involvement	15
Patients with severe uveitis	9

Patients presented with JIA-related involvement in other joints apart from the hip, with 24 patients experiencing knee involvement (18 of them bilaterally), 15 patients with ankle involvement, and small joint involvement in another 15 patients. Nine patients also had severe uveitis.

**Figure 1:**
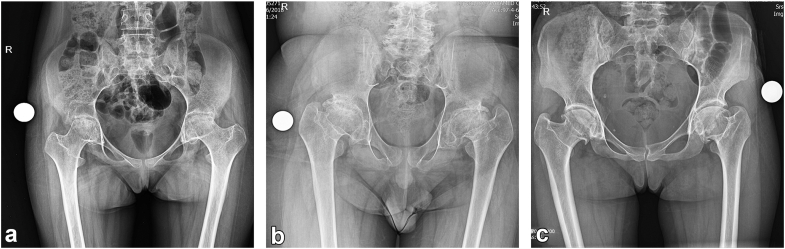
Preoperative anteroposterior pelvic X-rays of 3 patients: (a) a 17-year-old female, (b) a 16-year-old male, and (c) a 31-year-old female. Acetabular protrusion is evident in the left hip of case (a). Coxa profunda is observed in both hips of case (a) and the left hip of case (c). Coxa vara, trochanteric overgrowth, and a marked decrease in medial offset are present in both hips of case (b) and the left hip of case (c).

Radiographically, 27 hips exhibited acetabular protrusion, and relative overgrowth of greater trochanter and coxa vara was evident in 54 hips (Fig. 1). All patients had severe bilateral hip pain prior to surgery, which was successfully alleviated following the arthroplasty procedure. However, the involvement of other joints, particularly the knee, continued to cause discomfort in some patients.

**Figure 2:**
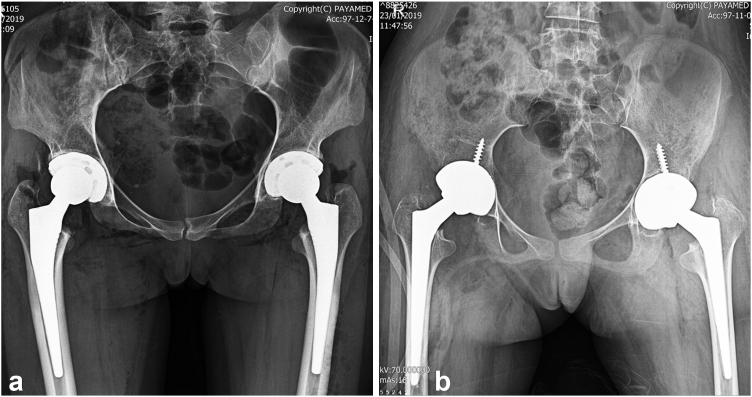
Postoperative anteroposterior pelvic X-rays of patient a (a) and patient c (b) from Figure 1, who underwent THA using a Pinnacle cup and Corail stem (Depuy Synthes) and a Continuum cup and Wagner cone stem (Zimmer Biomet), respectively.

Average intraoperative blood loss was 623 cc (range: 350-1100 cc), with 3 patients requiring allogenic blood transfusion. The mean operation time was 67 minutes per hip (range: 61-75), with an average interval of 9 minutes between surgeries (range 7-13). The total average operation time was 143 minutes (range 131-154). Patients stayed in the hospital for an average of 4.5 days (range: 3-6 days). Figure 2 shows the postoperative radiographs of the 2 patients whose preoperative X-rays are shown in Figure 1.

There was a significant functional improvement noted in all patients during the final follow-up. The mean preoperative HHS was 49.6 points (range: 39.1-84.3), which improved to 79.7 points (range: 61-98) postoperatively (*P* < .001). Postoperatively, all patients, except 3 requiring walkers due to knee involvement, were no longer dependent on assistive devices.

Preoperative flexion contractures were present in all hips, with a mean flexion contracture of 35 degrees (range: 10-75), mean adduction contracture of 7.5 degrees (range: 0-10), and mean arc of rotation of 15 degrees (range: 0-30). Postarthroplasty, there was a substantial improvement in the range of motion (ROM), with 27 patients showing no remaining contracture and 6 patients having residual contractures of 10° and 20° in one of included hips.

Concerning the use of disease-modifying antirheumatic drugs, all patients were on corticosteroids both at the time of surgery and at their most recent follow-up. In addition to corticosteroids, 10 patients were on methotrexate, 9 were on azathioprine, 3 utilized non-steroidal anti-inflammatory drugs, and 8 were on sulfasalazine during the time of their surgery. At the latest follow-up, only 1 patient was using a disease-modifying antirheumatic drug other than corticosteroids, specifically azathioprine. All of patients were in remission at the latest follow-up.

Complications were 5 instances (7.5%) of intraoperative fractures. Three patients sustained cracks in the calcar during reduction and 2 patients experiencing greater trochanter fractures during broaching, which were treated with wiring. Notably, the final HHS of these patients were comparable to those of other patients (HHS = 81.2; *P* > .05). There were no instances of dislocation, postoperative periprosthetic fracture, periprosthetic joint infection, aseptic loosening, or any revision surgery during follow-up until July 2022. One patient had a superficial wound dehiscence in the early postoperative period, which was successfully managed conservatively. Patients did not have any readmission in their follow-up.

## Discussion

This study provides compelling evidence that supports the safety of simultaneous bilateral THA performed via DAA in treating end-stage hip involvement due to JIA. The key outcomes we assessed were functional, radiological, patient-reported outcomes, and complications. Our results indicate a statistically significant improvement in functional outcomes as assessed by the HHS, alongside a commendable safety profile, and satisfactory patient-reported outcomes.

A unique strength of our study is the long-term follow-up of patients, which revealed a sustained improvement in the HHS, thus implying a durability of the THA in these patients. The mean HHS improved from 49.6 preoperatively to 79.7 postoperatively, emphasizing the efficacy of the procedure in drastically improving the quality of life for patients with JIA. This result is aligned with earlier studies that reported acceptable outcomes of THA in young patients with various types of hip diseases [[13], [14], [15]]. Another significant outcome of the study was the ability to alleviate pain in patients. All patients had severe bilateral hip pain prior to surgery, which was successfully ameliorated following the arthroplasty procedure.

**Figure 3:**
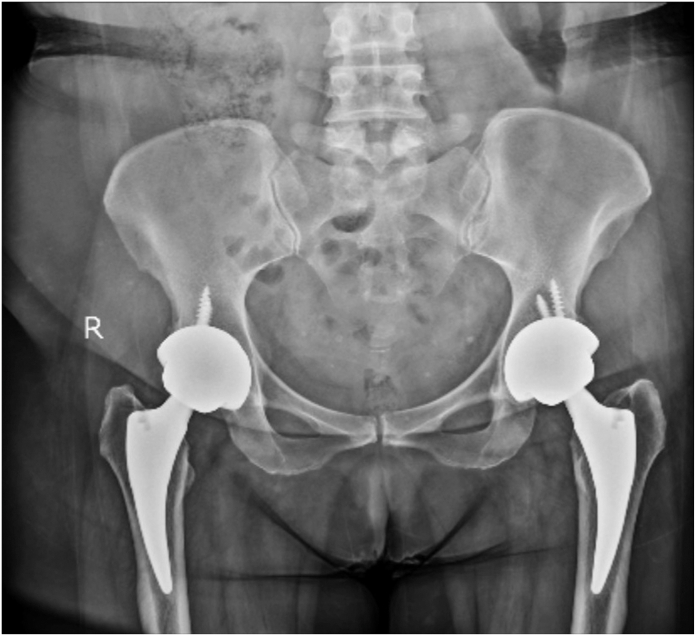
Pelvic anteroposterior X-ray taken at the 12-year follow-up of one of the patients undergoing simultaneous bilateral cementless THA using Continuum cup and Fitmore stem (Zimmer, Biomet).

The postoperative stability of both components was confirmed at the final follow-up (Fig. 3). Contrary to the findings in the study by Torchia et al., which reported early failures and loosening in some participants, such outcomes were absent in our patient cohort [16]. This aligns with the results presented by Restrepo et al. The tapered femoral components used can be press-fitted, minimizing the potential for fracture or subsequent subsidence [17]. The elevated failure rate of THA in younger patients has largely been ascribed to the heightened demands placed on the prosthetic hip and their increased activity levels [18]. Notably, these patients often diverge from other young patient cohorts in their functional demands and activity profiles due to extensive joint involvement and accompanying disabilities [19].

Although cemented fixation might seem advantageous for these individuals, Torchia et al.'s study, evaluating the outcomes of 63 cemented THAs in 50 adolescent patients, demonstrated that the likelihood of failure, whether in the form of revision or symptomatic loosening, progressively increased, culminating in a 45% rate after 15 years. Long-term data for cemented components indicate suboptimal survivorship, particularly concerning cemented cups [4,20,21]. Conversely, a consensus from various research endeavors advocates for cementless fixation as the superior choice for these patients [5,22,23].

One of the important issues that surgeons encounter when performing THA in patients with JIA is severe flexion contracture and nearly fused hips, as indicated by the ROM data in our results section [24]. Anterior capsulectomy is part of DAA in THA, besides that, theoretical advantages of anterior capsulectomy are improved exposure and ROM particular in external rotation and extension [10,24]. This aspect of the DAA is particularly beneficial for addressing the limited ROM seen in these patients.

Despite the overall positive outcomes, it is crucial to highlight the challenges faced during the study. Notably, 3 patients sustained calcar cracks and 2 experienced greater trochanter fractures. In an Insurance Claims Database study, Sequeira et al. found that patients with JIA experience more periprosthetic fractures (odds ratio = 2.93, *P* < .001) [25]. In another nationwide study by Schnaser et al. on complications of THA in 86,671 patients with inflammatory arthritis (2496 with JIA), it was found that patients with JIA had both the highest complication rate and the highest risk of periprosthetic fracture (odds ratio = 4.3, 95% confidence interval = 2.2-8.2) [26]. This increased risk is probably due to poor bone quality and impaired bone vascularity associated with JIA [27]. However, in our study, these complications were promptly managed with wiring, indicating that while complications may arise, they can be effectively managed without impacting the long-term results of the procedure. Also, one patient experienced a wound dehiscence in the early postoperative period, which was successfully treated conservatively.

It's worth noting that the study also brings to light the nonisolated nature of JIA, with a considerable proportion of patients experiencing JIA-related involvement in other joints apart from the hip [28]. While THA significantly alleviated hip pain, the involvement of other joints, particularly the knee, continued to cause discomfort in some patients. This finding underlines the need for an integrated multidisciplinary approach for the effective management of JIA.

Our study has several limitations that warrant consideration. First, the retrospective nature of our research introduces inherent challenges, such as potential biases in data collection and the possibility of missing or incomplete data. Additionally, the absence of a control group prevents us from drawing firmer conclusions. Furthermore, this study was conducted by a single surgeon, which may limit the variability of surgical techniques and outcomes observed. Over the 10-year period of the study, changes in postoperative protocols might also have influenced the results, yet these variations were controlled for in our study best as possible. Given that this research was performed in a tertiary referral hospital, the findings may not be generalizable to settings with less specialized surgeons or different patient demographics. These factors should be carefully considered when interpreting the results and applying them to broader populations.

## Conclusions

Our results suggest that simultaneous bilateral THA using DAA is a viable, effective, and safe surgical intervention for patients with JIA with bilateral end-stage hip involvement. This approach yields significant improvements in functional and radiological outcomes, with substantial pain reduction, while maintaining a favorable complication profile.

## Declaration of Generative AI and AI-Assisted Technologies in the Writing Process

We acknowledge the use of ChatGPT [https://chat.openai.com/] to edit our writing at the final stage of preparing our manuscript.

## Conflicts of Interest

The authors declare the following financial interests/personal relationships which may be considered as potential competing interests.

For full disclosure statements refer to https://doi.org/10.1016/j.artd.2024.101557.

## CRediT authorship contribution statement

**Mohammadreza Razzaghof:** Supervision, Data curation, Conceptualization. **Mohammad Vahedian Ardakani:** Data curation. **Mohammad Poursalehian:** Writing – review & editing, Writing – original draft. **Seyyed Hossein Shafiei:** Supervision. **Mahlisha Kazemi:** Data curation. **Seyed Mohammad Javad Mortazavi:** Writing – review & editing, Visualization, Validation, Conceptualization.

## Appendix A Supplementary data

Conflict of Interest Statement for Shafiei

Conflict of Interest Statement for Kazemi

Conflict of Interest Statement for Poursalehian

Conflict of Interest Statement for Razzaghof

Conflict of Interest Statement for Ardakani

Conflict of Interest Statement for Javad Mortazavi
